# Central Pontine and Extrapontine Myelinolysis: The Great Masquerader—An Autopsy Case Report

**DOI:** 10.1155/2014/745347

**Published:** 2014-03-04

**Authors:** Sajish Jacob, Harsh Gupta, Dejan Nikolic, Betul Gundogdu, Shirley Ong

**Affiliations:** ^1^Department of Neurology, Vanderbilt University, Nashville, TN, USA; ^2^Department of Neurology, University of Arkansas for Medical Sciences, Little Rock, AR, USA; ^3^Department of Pathology, University of Arkansas for Medical Sciences, Little Rock, AR, USA

## Abstract

Central pontine myelinolysis is a demyelinating disorder characterized by the loss of myelin in the center of the basis pontis usually caused by rapid correction of chronic hyponatremia. The clinical features vary depending on the extent of involvement. Demyelination can occur outside the pons as well and diagnosis can be challenging if both pontine and extrapontine areas are involved. We herein report a case of myelinolysis involving pons, lateral geniculate bodies, subependymal region, and spinal cord. To the best of our knowledge, this case represents the second case of spinal cord involvement in osmotic demyelination syndrome and the first case of involvement of thoracic region of spinal cord.

## 1. Introduction

Central pontine myelinolysis (CPM) is a demyelinating disorder characterized by the loss of myelin in the center of the basis pontis, usually caused by rapid correction of chronic hyponatremia. On rare occasions, demyelination occurs outside the pons and is termed extrapontine myelinolysis (EPM). The term osmotic demyelination syndrome (ODS) refers to demyelination caused by changes in serum osmolality and may result in both pontine and extrapontine myelinolysis. Known risk factors for this condition include alcoholism, malnutrition, systemic medical disease, liver transplantation, and rarely, hemodialysis [1–3]. We report an unusual case of widespread CPM and EPM affecting the brain and spinal cord in a man with no known risk factor to develop this condition other than fluid resuscitation.

## 2. Case Report

A previously healthy 74-year-old Caucasian male presented to our facility for evaluation of fluctuating mentation, vision loss, and lower extremity weakness of three months duration. Four months prior to admission, he had frequent bouts of nausea and vomiting, resulting in weight loss. Following an episode of severe hematemesis, he received fluid resuscitation and blood transfusion at an outside facility. An esophagogastroduodenoscopy revealed peptic ulcer disease.

A few days after his infusion, he became intermittently confused and started complaining of double vision. After a few days, he lost vision in his right eye and became profoundly weak on both legs. His left eye lost vision shortly after.

His neurologic examination showed a confused, awake, and elderly man. His vision was limited to light perception in his right eye and finger counting in his left eye. Strength in both lower extremities was 1-2/5. A sensory level to pin prick was noted below the T10 dermatome and vibration and proprioception were intact. He was areflexic and had a right extensor plantar response.

**Figure 1:**
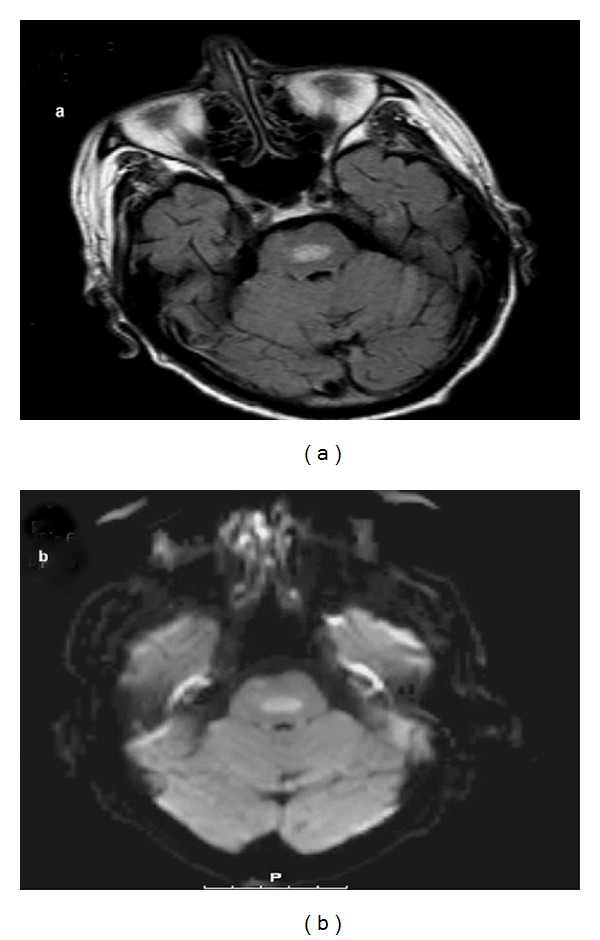
Ill-defined FLAIR hyperintense lesion seen in central pons (a) and restricted diffusion noticed in pons on DWI sequence (b).

**Figure 2:**
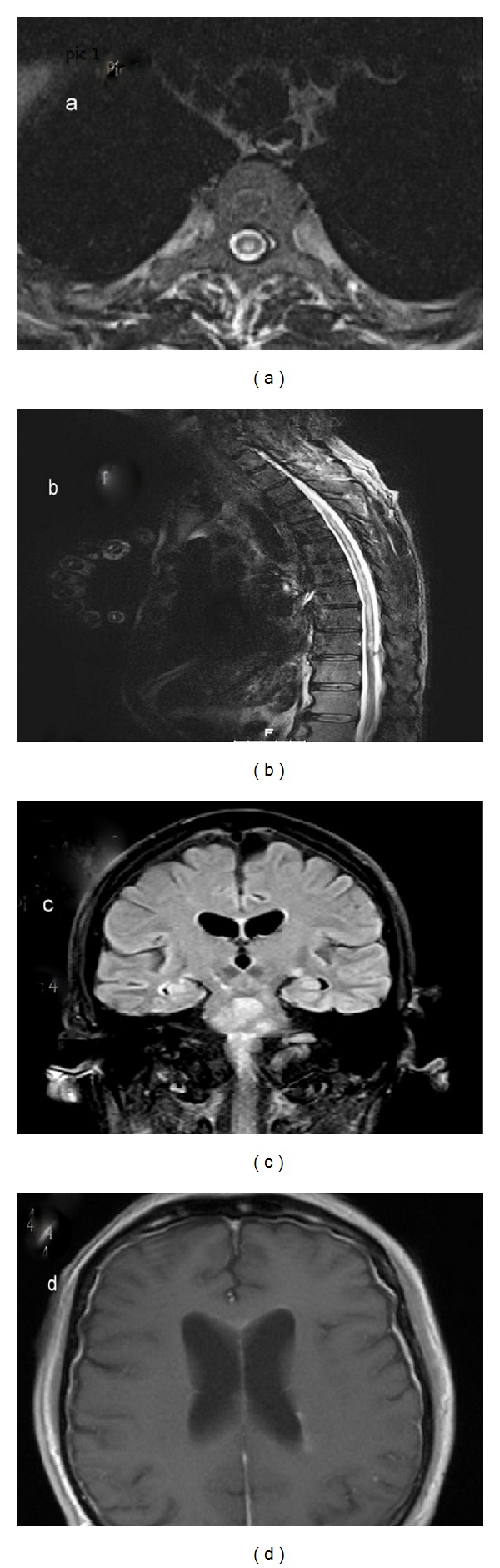
(a) Axial T2W of thoracic spinal cord and (b) sagittal T2W of thoracic spinal cord demonstrating intramedullary central T2 hyperintensity which extends from T2 to T10 level. (c) Coronal FLAIR demonstrating hyperintensity along the walls of the lateral and third ventricles, brainstem, and lateral geniculate bodies; on T1 postcontrast imaging (d) mild enhancement is noticed along the walls of the lateral ventricles.

Magnetic resonance imaging (MRI) of his brain and entire spine without and with contrast was performed. This study revealed fluid attenuated inversion recovery (FLAIR) hyperintense lesion in the pons, along the walls of the lateral and third ventricles, and in the brainstem. There was restricted diffusion in the pons. On T1 postcontrast imaging, mild enhancement was noticed along the walls of the lateral ventricles. MRI of the spine revealed intramedullary T2 hyperintensity extending from T2 to T10 level (Figures 1 and 2). MR spectroscopy of the pontine lesion revealed nonspecific findings. The differential diagnoses of these radiologic findings include CNS lymphoma, leptomeningeal metastatic disease, and atypical infection.

His initial complete blood count, liver function tests, kidney function tests, and electrolytes were all within normal limits except for mild anemia of 13.2gm/dL and mild hyponatremia of 134 meq/L. CSF opening pressure was normal. The CSF was clear in appearance with zero RBCs, 4 WBCs, protein of 143 mg/dL, and glucose of 58 mg/dL. Cytology did not show malignant cells, and flow cytometric analysis did not identify any abnormal lymphoid population. CSF oligoclonal bands, CSF VDRL, HSV PCR, CMV PCR, EBV PCR, enterovirus PCR, W. Nile IgG and IgM, bacterial, fungal, and AFB cultures, CSF cryptococcal antigen, and CSF ACE level were all unremarkable. CSF myelin basic protein was elevated at 12.90 ng/mL (range: 0.00–1.10 ng/mL). Vitamin B12, Thiamine, and serum NMO antibody titers were normal. CT scan of chest, abdomen, and pelvis was unremarkable. A whole body PET scan was performed, which did not reveal any abnormality.

Neurosurgery was consulted for a biopsy, but the lesions were considered inaccessible. The patient was empirically started on 3 days of methylprednisolone but did not result in any clinical improvement. Due to continued deterioration, he was discharged to hospice where he passed away two weeks later.

**Figure 3:**
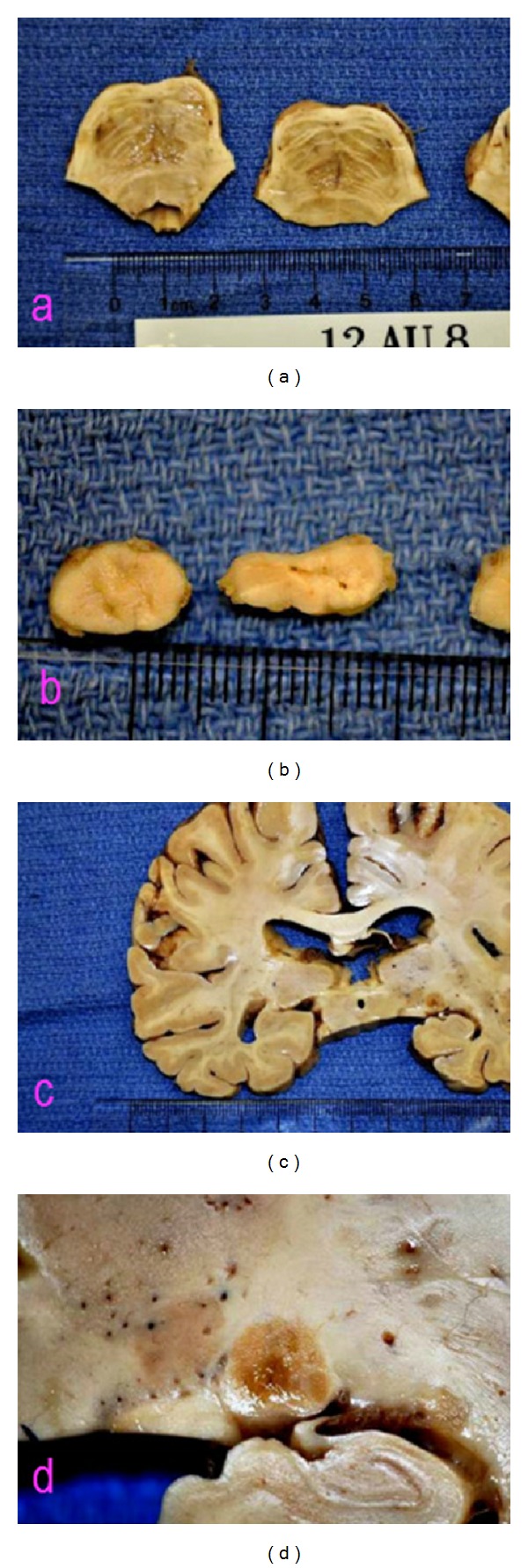
Coronal section of pons (a) demonstrating area of grey-tan softening enclosed peripherally by tissue of normal appearance; thoracic spinal cord section (b) demonstrating central grey area with granular appearance; coronal section of brain (c) demonstrating symmetrical grey-brown spots above temporal horns; and lateral geniculate body and subependymal area (d) with grey-brown and granular appearance.

**Figure 4:**
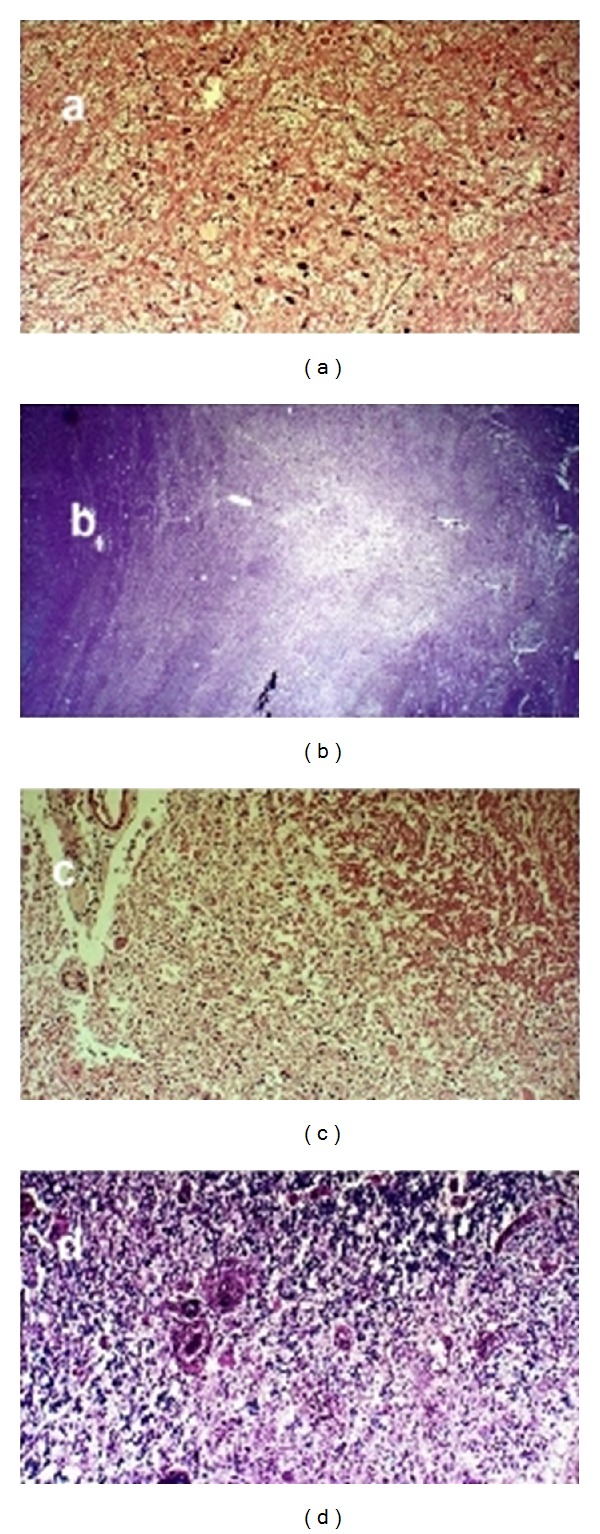
H and E staining of pons (a) shows neurons at the base well preserved against background of demyelinated area and LFB staining of pons (b) demonstrating demyelinated triangular shaped area in central portion of basis pontis. H and E staining of spinal cord (c) shows demyelination in central portion of spinal cord and well preserved fascicle on upper right and LFB staining of the cord (d) demonstrating demyelination of spinal cord and macrophages around blood vessels (left of center).

An autopsy was performed, revealing multiple areas of destructive lesions in the cerebrum, the lateral geniculate bodies, the caudate, both thalami, subependymal areas of the temporal horn of lateral, third, and fourth ventricles, the brain stem, and the spinal cord. The largest grossly identifiable lesion in the pons showed a roughly triangular shaped area of paleness, well demarcated from the surrounding tissue. Luxol fast blue combined with periodic acid schiff stain revealed pale staining, indicating a lack of myelin in the lesion. Immunohistochemical stain for neurofilament only demonstrated a mild to moderate loss of axonal fibers. The examination of the spinal cord revealed similar appearing lesions, mainly involving central regions of the cord, including gray matter. The lesion in the lateral geniculate bodies and subependymal regions also shared similar morphologic characteristics to the pons (Figures 3 and 4). These changes in spinal cord, lateral geniculate bodies, and subependymal regions were consistent with myelinolysis.

## 3. Discussion

Adams and colleagues first described CPM as an entity mostly affecting alcoholics and malnourished in 1959 [4]. In their description of 4 patients the pons was the only region implicated. It was eventually recognized that demyelinating lesions can also be seen outside the pons, termed EPM [1, 2]. In a report of 58 cases by Gocht and Colmant, the cerebellum and the lateral geniculate body were found to be the most commonly affected in EPM [5]. This study also revealed that EPM can occur in isolation or with CPM. Other sites that can be involved in EPM include external and extreme capsules, hippocampus, putamen, cerebral cortex/subcortex, thalamus, and caudate nucleus [1, 5].

Osmotic demyelination may occur in certain conditions such as alcoholism, malnutrition, after prolonged diuretic use, prolonged vomiting, burns, chronic psychogenic polydipsia, after liver transplant, and rarely, after pituitary surgery and after urological surgery/gynecological surgery, especially those requiring glycine infusions [1]. ODS has also been reported in hemodialysis patients [3]. Factors putting patients at risk for osmotic demyelination include a low serum sodium at presentation (120 meq/L or less), prolonged hyponatremia, and rapid rate of correction. Although there is no safe rate of correction, the greatest risk appears to be when serum sodium was corrected by more than 20 meq/L in 24 hours [6–8].

Symptoms of CPM are typically biphasic. Initial hyponatremia causes encephalopathy or seizures with symptoms of CPM being delayed for two to six days after correction of hyponatremia [4, 7]. Clinical symptoms and signs of CPM may include dysarthria, dysphagia, quadriparesis, oculomotor abnormalities, and locked-in-syndrome depending on the site of involvement in the pons [1]. Different types of movement disorders have also been described in EPM, including mutism, parkinsonism, catatonia, dystonia, and tremors [1]. The clinical diagnosis can be challenging if CPM occurs in conjunction with EPM like it did in our patient.

Detection of ODS with the help of imaging is superior with an MRI of the brain than with a computed tomography scan. Findings on the MRI can develop as late as 4 weeks after the onset of symptoms, and imaging findings can even be present in asymptomatic individuals [9]. MRI findings in ODS usually shows symmetric signal hyperintensity in the central pons on T2 weighted and FLAIR imaging. Diffusion weighted imaging can detect changes of ODS before FLAIR and T2 sequences [10]. Pathologically, ODS is characterized by sparing of axons and neurons, sparse or absent infiltration by lymphocytes, and degeneration or loss of oligodendrocytes [1, 11].

There were some unusual features in our case; namely, the patient did not have a history of alcoholism, liver disease, severe electrolyte disturbances, or any other major medical co-morbidities prior to his neurological deterioration. The most likely cause of osmotic demyelination was the aggressive correction with fluids when he had a bout of hematemesis. In our literature review, we found one case report of cervical cord involvement in ODS in a patient with pancreatitis [12] and another case of cervical spinal cord involvement without pathological findings supporting it [13]. As far as we are aware, our case represents the second case of spinal cord involvement in ODS. The subependymal nature of involvement and widespread central involvement of the spinal cord in our case is unique.

This case demonstrates the need to consider EPM as a differential diagnosis in cases where patients are at risk to develop ODS and their imaging suggests neurological damage affecting different parts of the brain and spinal cord, since ODS damage may not necessarily just be limited to the pons.
